# Assessment of water, sanitation and hygiene services within nineteen Rohingya camps in Cox’s Bazar, Bangladesh in 2022

**DOI:** 10.1186/s12913-025-12874-8

**Published:** 2025-06-09

**Authors:** Saira Butt, Md. Samiur Rahman Chowdhury, Sohana Sadique, Abdullah Al Faisal, Adrien Mahama, Alexander Gorski, Biserka Pop-Stefanija, David Beversluis, Jackson Mojong Lochokon, Kalyan Velivela, Kennedy Uadiale, Md. Mahbubur Rahman, Teju Gebeyaw Worku, Thok Johnson Gony Billiew, Patrick Keating

**Affiliations:** 1 Médecins Sans Frontières, Cox’s Bazar, Bangladesh; 2 Médecins Sans Frontières, Dhaka, Bangladesh; 3 Department of Earth and Planetary Sciences, McGill University, Montreal, Canada; 4 Médecins Sans Frontières Intersection, Dhaka, Bangladesh; 5 Médecins Sans Frontières, Amsterdam, The Netherlands; 6 Médecins Sans Frontières, Nairobi, Kenya; 7 Civil Surgeon, Cox’s Bazar, Bangladesh; 8 Médecins Sans Frontières, London, UK

**Keywords:** Refugee, Water, Sanitation, Hygiene, Rohingya, Survey, Diarrhoea, Skin infection

## Abstract

**Background:**

Since August 2017, approximately 960,000 Rohingya refugees have settled in Cox’s Bazar, Bangladesh. Water, sanitation, and hygiene (WASH) infrastructure and programs were implemented across the camps to address the needs of the population and reduce the burden of linked infectious diseases. However, monitoring and maintenance of this infrastructure has been inconsistent. This study aimed to assess progress in WASH in the camps of Cox’s Bazar since the early emergency phase in 2018, and to update the priorities for intervention.

**Methods:**

From January to March 2022, a lot quality assurance sampling (LQAS) survey was conducted across 19 camps. Nineteen households were randomly selected per camp. Data on access to and quality of WASH services, household practices, and health outcomes including skin infections among children under five years of age were collected. Crude and weighted averages with 95% confidence intervals were calculated for each indicator and compared with targets pre-defined based on Sphere guidelines and Médecins Sans Frontières WASH experts. Chi-squared tests were used to compare the results to a 2018 LQAS survey.

**Results:**

More than half of the indicators (59%; 16/27) did not meet the pre-determined targets. Performance was adequate on three of five water quality and supply indicators, with less than half of households (44%, 95% CI: 39–49%) reporting that water was continuously available in the past week. Regarding water storage, performance on three indicators was considered adequate, as the proportion of households that keep water for less than one day was 27% (95% CI: 23-32%). Of six hygiene indicators, adequate performance was identified for only one. Performance on the sanitation indicators was inadequate, with 11% (95% CI: 8-15%) of households using an improved sanitation facility. In solid waste management, two of four indicators suggested adequate performance, and for health outcomes, the proportion of children who hadn’t shown any skin infection was inadequate at 69% (95% CI: 64-73%).

**Conclusions:**

Improvements in the WASH situation in Cox’s Bazar have been observed in 2022 compared to 2018. However, significant gaps remain in water supply, sanitation facilities, and hygiene services. LQAS can be an effective monitoring tool to support long-term multisectoral interventions in protracted emergencies.

**Supplementary Information:**

The online version contains supplementary material available at 10.1186/s12913-025-12874-8.

## Background

In Cox’s Bazar, Bangladesh, a population of nearly 900,000 Rohingya, a minority ethnic group persecuted in Myanmar, live in refugee camps [1]. While Rohingya refugees have been living in Bangladesh since 1978, in August 2017 heightened ethnic and religious persecution in Myanmar led to more than 700,000 people fleeing to Cox’s Bazar in the space of a few months [1]. This mass migration led to a rapid effort to provide access to water, sanitation, and hygiene (WASH) services. By March 2018, more than 6,000 water points and 50,000 emergency latrines had been built [2]. As the WASH sector began adapting to the prolonged situation, efforts shifted towards building piped water networks and faecal sludge treatment plants [3]. However, challenges remain with maintaining WASH standards. A study in 2019 revealed faecal coliform contamination in 28% of samples from tubewells, and 74% contamination at point-of-use [4]. A 2021 report indicated that 22% of sanitation facilities required maintenance, and highlighted risks of gender-based violence associated with accessing sanitation facilities [3]. Inadequate WASH can result in gastrointestinal, skin, and eye infections, particularly affecting children [5–7]. Furthermore, menstrual hygiene has been a low priority in this setting, with women and girls reporting having inadequate materials and facilities to manage menstruation with dignity and with low risk of infection [3, 8]. 

Routine monitoring can support the maintenance of WASH facilities and services, improving living conditions and consequently public health. Lot quality assurance sampling (LQAS) surveys can be used for routine monitoring of WASH services. LQAS methods were originally developed to provide assessments of manufacturing quality and have since been adapted to the humanitarian context for rapid assessments of vaccination coverage, prevalence of disease, and water and sanitation conditions [9, 10]. Further studies have suggested its use as a tool for disease surveillance, monitoring and evaluation of public health programs [11–14]. 

In this study, LQAS is presented as a monitoring tool for WASH services in the refugee camps in Cox’s Bazar, Bangladesh. After the emergency response phase began, a LQAS survey was conducted in 2018 to assess gaps in WASH services. However, as the humanitarian emergency has become prolonged and aid has begun to decrease, there is a need to monitor and quantify deterioration, and to prioritise areas with the greatest deterioration. Failing to do so may increase refugees’ exposure to health hazards that could increase mortality and morbidity. In this paper we present the results of a second LQAS survey conducted in 2022 and propose the use of LQAS as a monitoring tool in low-resource, prolonged emergency situations where consistent and reliable data on WASH service provision are not available.

By comparing the results of LQAS surveys conducted four years apart, we describe and quantify changes in the quality, availability, and accessibility of WASH services from the initial rapid response stage of an emergency to a long-term humanitarian situation.

## Methods

### Study setting

**Fig. 1:**
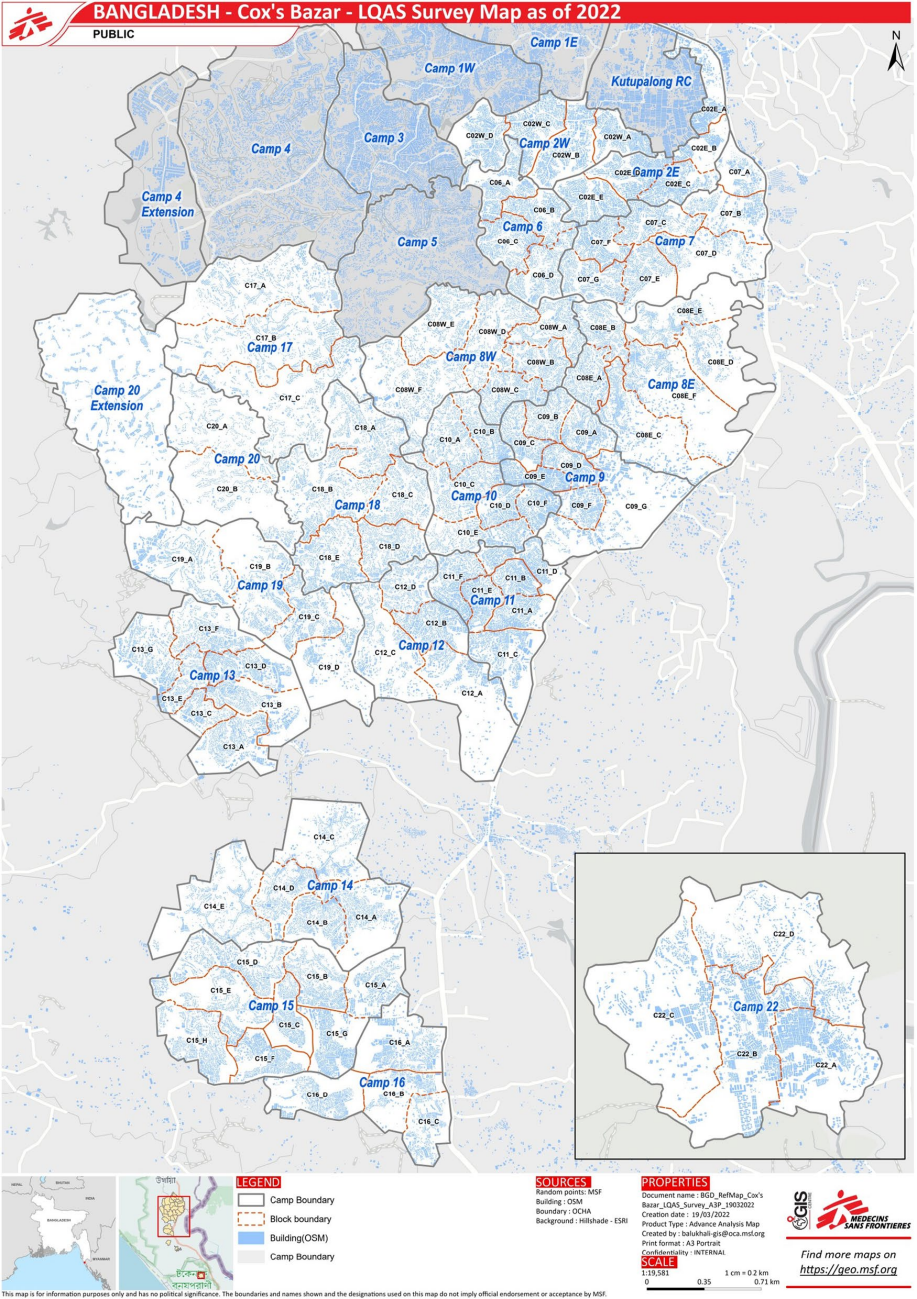
Map of Cox’s Bazar camps with surveyed camps and blocks highlighted

In 2022, there were more than 900,000 Rohingya people living in the refugee camps of Cox’s Bazar, Bangladesh, of which 52% are female and 52% are children aged 1 to 17 years [15]. There are 34 camps, located in the Ukhiya and Teknaf Upazilas of Cox’s Bazar. Each camp is divided into multiple blocks (Fig. 1).

### Study design

A cross-sectional LQAS survey was conducted from January to March 2022 in 19 camps. Fifteen of the 19 camps were also included in a 2018 survey on 21 camps that used the same methodology and used many of the same questions. LQAS are commonly used to classify and prioritise needs at smaller geographic levels, called supervision areas (SAs), which are areas at which interventions can make programmatic sense [9, 16]. For efficiency, LQAS often use a sample size of 19 units per SA, and then aggregate the data to calculate coverage proportions for the entire catchment area with 95% confidence intervals [16]. These proportions are compared to pre-determined decision rules (DRs) to define adequate or inadequate performance for each SA on each indicator, and then overall on each indicator [16]. In this study, camps were defined as SAs and expected target values were pre-defined for each indicator based on Sphere guidelines [17] or the experience of the Médecins Sans Frontières (MSF) Water and Sanitation team. These pre-defined target values can be found in the Annex Table 1.

### Sampling

The study population included households in the 19 camps, comprising a total of 534,356 individuals [18]. Two distinct “universes” were sampled in the study. The first universe comprised all households in the camps, while the second universe consisted of parents or guardians of children under the age of five who resided in households selected for the first universe. A sample size of 19 households and 19 parents or guardians of children aged under five years was selected for each camp to maintain α (probability of misclassifying an area with high coverage as “low”) and β (probability of misclassifying an area with low coverage as “high”) errors at < = 10%, resulting in a sample size of 361 for each universe.

Within each camp, probability proportional to size sampling (PPS) was used to calculate the number of households to be recruited in each block based on population size, using the LQAS Generic Toolkit [16]. The R package “rgdal” generated the calculated number of geospatially random points of x-y coordinates within each block. The 19 selected points from each camp were then imported into the offline Open Street Map Automated Navigation Directions (OsmAnd) maps mobile application for data collection. Once at the specified coordinates, enumerators approached the nearest household for recruitment.

### Data collection

A structured questionnaire was used to gather information on WASH and waste management practices, as well as the incidence of WASH-related illnesses including diarrhoea, skin and eye infection, and jaundice among children below five years old. The questionnaire was adapted from one previously used in other MSF programs [9]. The data collectors received two days of training in the local language of Rohingya-Chittagonian. Before initiating the survey, a one-day pilot was conducted to ensure acceptability of questions and enumerators’ understanding of data collection practices. Data was collected using the KoBoCollect application on mobile phones.

The selection of households and respondents involved identifying an individual over the age of 18 who had adequate knowledge of the household behaviours and practices. If such an individual was unavailable or refused to participate, the survey team proceeded to the nearest household to the left. Additionally, one child under five years of age was included from the same household, and WASH-related diseases over the prior two weeks were assessed. In situations where there were multiple under-five children in the household, one child was selected using the random selector part of the KoboCollect application used to collect data. In cases where no such child was available, the survey team continued to the next nearest household to the left until a parent or guardian of a child younger than five years old was identified.

### Data analysis

Data were imported into the Liverpool School of Tropical Medicine’s (LSTM) Generic Household Survey Tools [16]. The tool was then used to aggregate data from all camps and to determine coverage indicators for the entire 19 camps. Weighted average coverage was calculated for each indicator across the 19 camps, by multiplying the crude proportion of positive responses for each camp by the proportion of the population that the camp represented in the survey, and then summing up. For each indicator, 95% confidence intervals (CI) were also calculated. A weighted value meeting the target DR was classified as adequate while a value lower than the target was considered inadequate.

At the camp level, those for which the crude averages for a given indicator were below both the target DR and the weighted average DR across all camps were considered high priority for intervention. Camps whose crude averages for a given indicator were below the target DR but met or exceeded the weighted average DR across all camps were considered medium priority. Camps whose crude average DRs for an indicator exceeded the target were considered low priority.

Additionally, a comparison was conducted between this LQAS survey and the previous LQAS survey performed in 2018, as there was an overlap in camps covered and indicators collected. Chi-squared tests were conducted on the weighted averages of the common indicators to compare the proportions for each indicator in 2018 to that of 2022 to understand if there was a significant change in those proportions over those time periods. Microsoft Excel 2016 and R version 4.1.2 were used to analyse the data.

## Results

Three hundred and sixty-one households participated in the survey, and the response rate was 100%. Male-headed households made up the majority (76%), with most of survey respondents being female (59%) and the median household size being six members.

Among water supply and quality indicators, adequate performance was reported for three out of five indicators, two of which showed improvement compared to the 2018 survey. The proportion of households using an improved water source for drinking water was measured at 53% (95% CI: 46-59%) in 2018 and rose to 99% (95% CI: 98-100%) in 2022. Similarly, the proportion of households reporting that the taste of the water from the nearest improved source is acceptable rose from 86% (95% CI: 83-90%) to 93% (95% CI: 90–96%). However, use of the same improved water source for all activities dropped from 99% (95% CI: 98-100%) to 91% (88-93%). Avoidance of using surface water for drinking and cooking and the proportion of households reporting that water was continuously available from habitual sources remained consistently below the target values in both 2018 and 2022, at 84% (95% CI: 82-87%) and 44% (95% CI: 39-49%), respectively in 2022 (Table 1).

**Table 1:** Water supply and quality coverage indicators in Cox’s Bazar, Bangladesh 2018 & 2022

Water supply indicator	Target coverage (%)	2018			2022			Status in 2022	Status of change from 2018–2022
		*n*	*N*	Weighted average (95% CI)	*n*	*N*	Weighted average (95% CI)		
Proportion of households that use an improved water source for drinking	95%	211	399	53% (46-59%)	359	361	99% (98-100%)	Adequate	Improved^*^
Proportion of households that report the taste of the water from the nearest improved water source is acceptable	80%	342	397	86% (83-90%)	337	361	93% (90-96%)	Adequate	Improved^*^
Proportion of households that use the same improved water source for all activities	90%	391	399	99% (98-100%)	319	361	91% (88-93%)	Adequate	Deteriorated^*^
Proportion of households that DO NOT report using surface water for drinking or cooking	95%	316	399	83% (81-86%)	304	361	84% (82-87%)	Inadequate	Stable
Proportion of households who report that water was continuously available from their habitual water source for the last week	80%	180	399	46% (41-51%)	161	359	44% (39-49%)	Inadequate	Stable

^*^Pearson’s Chi square test with a *p*-value of < 0.05

Of five water storage indicators, performance was deemed to be adequate on three: The proportion of households treating their water increased from 43% (95% CI: 39-48%) in 2018 to 78% (73-82%), and the proportion of households with water containers of at least 10 L-capacity and those cleaning the container at least once a week remained stable, at 96% (95% CI: 94-99%) and 92% (95% CI: 89-95%) respectively in 2022. However, a reduction was observed in the proportion of households reporting they kept water in their homes for less than one day, from 97% (95% CI: 95-98%) in 2018 to 27% (95% CI: 23-32%) in 2022 (Table 2).

**Table 2:** Water storage indicators in Cox’s Bazar, Bangladesh, 2018 & 2022

**Water storage indicator**	**Target coverage (%)**	2018			2022			Status in 2022	Status of change from 2018–2022
		***n***	***N***	**Weighted average** **(95% CI)**	***n***	***N***	**Weighted average** **(95% CI)**		
Proportion of households that have water containers of at least 10 L total capacity	90%	374	399	94% (91-96%)	350	361	96% (94-99%)	Adequate	Stable
Proportion of households that clean the inside of water containers at least once a week	80%	393	399	99% (97-100%)	327	353	92% (89-95%)	Adequate	Deteriorated^*^
Proportion of households that keep water in household containers for less than one day	90%	385	399	97% (95-98%)	90	353	27% (23-32%)	Inadequate	Deteriorated^*^
Proportion of households that find the taste of chlorinated water to be acceptable	80%	169	290	58% (52-64%)	274	361	74% (70-79%)	Inadequate	Improved^*^
Proportion of households whose water was treated with chlorine, either tablet (Aquatabs) or at the point of collection when they last collected drinking water	80%	176	399	43% (39-48%)	286	361	78% (73-82%)	Adequate	Improved^*^

^*^Pearson’s Chi square test with a *p*-value of < 0.05

On the other hand, adequate performance was detected on only one of the six hygiene indicators: 75% (95% CI: 71-80%) of households reported having been visited by a hygiene promoter in the previous week, which remained stable since 2018. The proportion of households with soap and the proportion of households whose female members use acceptable materials for menstrual hygiene both deteriorated, from 98% (95% CI: 97-99%) to 91% (95% CI: 89-94%) and from 98% (95% CI: 96-99%) to 62% (95% CI: 57-66%) respectively (Table 3). In 2022 the additional indicators on menstrual hygiene revealed further inadequate performance, with 74% (95% CI: 71-77%) of households having received menstrual hygiene products from a distribution and 87% (95% CI: 83-91% reporting appropriate disposal of single use menstrual hygiene products; however, only one-third of households responded to this question.

**Table 3:** Hygiene practice and coverage indicators in Cox’s Bazar, Bangladesh, 2018 & 2022

Hygiene indicator	Target coverage (%)	2018			2022			Status in 2022	Status of change from 2018–2022
		*n*	*N*	Weighted average (95% CI)	*n*	*N*	Weighted average (95% CI)		
Proportion of households that can show at least one piece of soap	95%	392	399	98% (97-99%)	330	359	91% (89-94%)	Inadequate	Deteriorated^*^
Proportion of households that currently have soap and water available for handwashing in the household	90%	332	399	83% (79-87%)	304	359	83% (80-87%)	Inadequate	Stable
Proportion of households that have been visited by a hygiene promoter within last week	80%	319	399	81% (77-85%)	280	361	75% (71-80%)	Adequate	Stable
Proportion of households whose female members use acceptable materials for menstrual hygiene	95%	381	389	98% (96-99%)	219	344	62% (57-66%)	Inadequate	Deteriorated^*^
Proportion of households that has ever received menstrual hygiene products from a distribution	95%	Not collected	Not applicable	Not applicable	259	344	74% (71-77%)	Inadequate	NA
Proportion of households that dispose of single use menstrual hygiene products appropriately	95%	Not collected	Not applicable	Not applicable	112	123	87% (83-91%)	Inadequate	NA

^*^Pearson’s Chi square test with a *p*-value of < 0.05

Based on sanitation indicators, performance was inadequate and remain unchanged compared with the 2018 survey. Approximately 10% of respondents among both men and women reported using latrines meeting improved sanitation facility criteria (based on having a functional lockable door, latrine not overflowing (24%), no visible faeces (42%), within 50 steps of the households and handwashing point with soap and water). In terms of child faeces disposal, more than a quarter of respondents reported disposing of them outside of the latrine (Table 4).

**Table 4:** Sanitation indicators coverage in Cox’s Bazar, Bangladesh, 2018 & 2022

Sanitation indicator	Target coverage (%)	2018			2022			Status in 2022	Status of change from 2018–2022
		*n*	*N*	Weighted average (95% CI)	*n*	*N*	Weighted average (95% CI)		
Proportion of households whose male members use an improved sanitation facility with an acceptable handwashing area that has soap and water point	90%	40	399	8% (6-10%)	42	361	11% (8-15%)	Inadequate	Stable
Proportion of households whose female members use an improved sanitation facility with an acceptable handwashing area that has soap and water point	90%	38	399	8% (5-10%)	41	360	11% (8-14%)	Inadequate	Stable
Proportion of households that dispose of children’s and babies’ faeces in an appropriate manner	95%	246	334	72% (67-77%)	244	361	72% (66-75%)	Inadequate	Stable

Pearson’s Chi square test with a *p*-value of < 0.05

On two out of five solid waste management indicators, performance was considered adequate. A quarter of respondents reported having at least a bucket/bin of 20 L, which was 45% below the target value. More than three quarters of respondents reported disposing their waste via communal waste collection (Table 5).

**Table 5:** Solid waste management indicators in Cox’s Bazar, Bangladesh, 2018 & 2022

Solid waste management indicator	Target coverage (%)	2018			2022			Status in 2022	Status of change from 2018–2022
		*n*	*N*	Weighted average (95% CI)	*n*	*N*	Weighted average (95% CI)		
Proportion of households that dispose of their waste via communal waste collection	80%	Not collected	Not applicable	Not applicable	309	361	86% (83-89%)	Adequate	NA
Proportion of household which are satisfied with the collection frequency of the waste by communal services	80%	Not collected	Not applicable	Not applicable	302	309	98% (96-99%)	Adequate	NA
Proportion of households for which specific types of waste are collected for reuse, recycling	50%	Not collected	Not applicable	Not applicable	118	361	30% (26-34%)	Inadequate	NA
Proportion of households that has at least 1 bucket/garbage bin of 20 L for solid waste storage	70%	Not collected	Not applicable	Not applicable	89	361	25% (21-29%)	Inadequate	NA

Pearson’s Chi square test with a *p*-value of < 0.05

Of the four disease indicators, adequate performance was reported on three; the skin infection indicator was the only exception. A fifth of children under 5 years were reported to have had diarrhoea in the two weeks preceding the survey and 31% had a skin infection in the same period. The proportion of children under 5 years with skin infection was much higher in 2022 compared to 2018 (Table 6). The performance of each camp per indicator can be found in the annex.

**Table 6:** Disease prevalence indicators in Cox’s Bazar, Bangladesh, 2018 & 2022

Disease indicator	Target coverage (%)	2018			2022			Status in 2022	Status of change from 2018-2022
		*n*	*N*	Weighted average (95% CI)	*n*	N	Weighted average (95% CI)		
Proportion of households reporting NOT HAVING diarrhoea among children <5 years in the last two weeks	80%	322	399	80%(76%-84%)	284	361	78%(74%-83%)	Adequate	Stable
Proportion of households reporting NOT HAVING eye infections among children <5 years in the last two weeks	80%	388	399	98%(96%-99%)	350	361	96%(94%-99%)	Adequate	Stable
Proportion of households reporting NOT HAVING skin infections among children <5 years in the last two weeks	80%	373	399	93%(91%-96%)	246	361	69%(64%-73%)	Inadequate	Deteriorated*
Proportion of households reporting NOT HAVING jaundice among children <5 years in last two weeks	80%	398	399	100%(99%-100%)	350	361	93%(92%-94%)	Adequate	Deteriorated*

^*^Pearson’s Chi square test with a *p*-value of <0.05

## Discussion

Provision and maintenance of WASH facilities are basic requirements for life and for maintaining the dignity for all populations, particularly for refugee populations in protracted crises such as the Rohingya living in Cox’s Bazar. Access to clean water in sufficient quantity and quality is a fundamental human right and is necessary to prevent illness and maintain general hygiene. This study found that 16 out of 27 WASH-related indicators did not meet the pre-determined targets, highlighting the vulnerability of this population to WASH-related morbidity and mortality.

### Water supply and storage

Adequate access to an improved water source was reported by nearly 100% of surveyed households, in line with earlier reports from United Nations High Commission for Refugees (UNHCR) in Cox’s Bazar and in other refugee camps in Uganda, Kenya and South Sudan [19]. The large improvements in access to improved water source between 2018 and 2022 likely reflect the transition from more limited availability of water services earlier in the emergency response compared to the current more stable context. However, no such improvements over time were observed in water supply where low levels of continuously available water were reported by households in 2018 and 2022. Water supply shortages have been reported in other refugee camps where most did not reach the minimum Sphere requirements of 20 L per person per day [17, 19–21]. These chronic water supply issues may also be linked to the decrease in households keeping water for less than one day and the higher levels of scabies in 2018 compared to 2022, as inconsistency in supply may lead to households rationing water. Previous reports have noted breakdown of handpumps, as well as limited groundwater availability during the dry season from November to June, particularly in Teknaf [22–24]. However, hydrogeological monitoring and modelling of these groundwater systems by MSF and others currently show that extraction rates have not exceeded sustainable levels [25]. Instead, poor operation and maintenance of water infrastructure are the principle cause of water disruptions [26–28]. 

### Hygiene

Compared to 2018, households were less likely to be able to show a piece of soap and to use acceptable materials for menstrual hygiene (disposable or reusable cloth/ sanitary pad). These results suggest a lack of access to hygiene materials, with such access issues being reported previously across Cox’s Bazar and other refugee camps [19, 29]. Similar issues with disposal of menstrual hygiene products have also been identified in an internally displaced population in Nigeria [30]. However, there is a higher degree of uncertainty on these questions as only 123 of 361 households responded to the question on disposal of menstrual hygiene products. Innovative approaches for the disposal of menstrual hygiene products have been reported in Cox’s Bazar, and the variations of this indicator in this survey at camp level may be explained by a limited scaling up of these approaches to date [31]. The poor performance of the hygiene indicators may also be linked to the limited water supply and the higher levels of scabies reported among children under 5 years reported in 2022. As scabies control requires washing clothes and bedding [32], shortages in water and soap can lead to higher prevalence of scabies as reported in other studies [33, 34]. 

### Sanitation management

Similar to other refugee camps, the sanitation situation in Cox’s Bazar was far below acceptable levels [19, 20]. Moreover, there has been no major change in access to an improved sanitation facility between 2018 and 2022 in Cox’s Bazar. The impact of the COVID-19 pandemic, fires, and a reduction in humanitarian aid from major funders such as the United Kingdom [35, 36] resulted in damage to and decrease in WASH activities, such as rehabilitation and construction of latrines and waste disposal [37–39]. The poor access to an improved sanitation facility reported in this study contrasts with a recent UNHCR study [19] on camps in Cox’s Bazar, but this is due to the inclusion of more criteria in this study’s definition of an improved sanitation facility (including distance from household, availability of soap and handwashing station, lockable door and no visible faeces around the latrine). Sphere guidelines suggest that there should be no more than 20 people per latrine [17, 40]. But in reality, this number may be higher in some camps, for example, closer to 30 people per latrine [40]. This higher person-to-latrine ratio could negatively impact cleanliness and increase risk of disease [17, 40]. 

### Waste disposal and recycling

Due to the densely populated nature of the Cox’s Bazar refugee camps, waste management and disposal have been challenging. Generally, most respondents from the 19 camps were satisfied with the collection frequency of the community waste disposal system. But there were variations at camp level, which have been reported in a previous study in refugee camps including those in Cox’s Bazar [19]. Community waste zones which are not appropriately managed can be breeding areas for rodents, flies and mosquitoes. This therefore increases the risk for vector borne diseases like dengue and malaria. For example, large dengue outbreaks occurred in Cox’s Bazar in 2022 and 2023 [41, 42]. 

### Health implications

A variety of WASH-related diseases have been widely reported in refugee camps [21]. Indicators of outbreak-prone diseases such as diarrhoea, eye infections, and acute jaundice syndrome were at acceptable levels among young children in 2022, showing little change from 2018, and aligning with ongoing facility-based and community-based surveillance. However, approximately 30% of households reported skin infections in the previous two weeks preceding our survey. This could be explained by the fact that an acceptable number of households are using improved water sources, which could reduce transmission of diarrhoea and jaundice. However, access to soap has deteriorated, which could exacerbate transmission of skin diseases.

These results were also validated by facility-based surveillance. During the survey period, there was an outbreak of scabies among the camps [43, 44]. The results of the LQAS survey were used with facility-based surveillance to provide an approximate estimate of scabies prevalence in the camps, providing adequate evidence to alert stakeholders and quantify the excess morbidity before a more comprehensive prevalence survey was implemented and used for decision-making regarding mass drug administration.

### Next steps

This LQAS survey highlighted several gaps in WASH service provision, at multiple levels, in Cox’s Bazar. To address these wide, and in some cases, chronic issues, a multi-faceted and integrated approach is required both in terms of infrastructure and behavioural elements. A continuous water supply needs to be ensured for this population, which requires that the existing water networks are regularly monitored, repaired and maintained. The long-term sustainability of the water aquifer below the camps in Cox’s Bazar must also be ensured. Other key priorities for the WASH sector include ensuring the population has enough soap, menstrual hygiene products, household waste containers and access to improved latrines and handwashing points. Adapting WASH-related health promotion messaging approaches to the population may also provide benefits, as gaps in knowledge and practices among some Cox’s Bazar and other refugee camp populations have been previously reported [29, 45, 46]. However, the usefulness of such activities will be influenced by whether the structural issues such as sufficient access to clean water and improved sanitation facilities have been addressed. Finally, routine use of LQAS surveys may be useful in facilitating ongoing monitoring of WASH challenges and successes and contribute to ensuring accountability of WASH actors for their activities.

### Strengths and limitations

In this paper we have compared the LQAS survey results from 2018 with those from 2022. The comparison can inform decision-making, enable stakeholders to track progress in WASH service delivery, and foster accountability in achieving goals and objectives. It provides insights for strategic planning and assessing performance status over the years. In addition, we employed PPS sampling in our study to mitigate sampling bias. The LQAS survey findings were triangulated with health facility and community-based surveillance data where applicable. Moreover, this survey complemented ongoing health facility-based surveillance, as it was able to capture the prevalence of WASH-related disease regardless of severity and access to healthcare. Cases with self-limiting diarrhoea or skin disease are less likely to present to healthcare facilities, especially considering barriers such as cost of travel and accompaniment, waiting times, and security checkpoints.

The findings also need to be considered in light of the limitations of the survey. Firstly, most of the questions were self-reported at household level, which may have introduced some social desirability bias as well as recall bias for questions involving events in the last two weeks. The survey was generally focused on quality and coverage of WASH services, while the knowledge, attitude and use of the services were not assessed. Additionally, assessment of water quality was limited as microbiological assessments were not included. On the menstrual hygiene questions, as less than one third of households responded, the indicators could not be assessed for many camps. The low response rate could be due to the taboo associated with menstruation in the Rohingya community [3]. In terms of assessing change, the study did not permit an assessment of the change in waste management indicators, as they had not been included in the 2018 survey. In addition, while there were 15 camps in common in both the 2018 and 2022 surveys, the comparison of the two surveys may be affected by those that were different. As with all LQAS surveys, the small sample size at supervision level can sometimes lead to incorrect classifications. But whenever possible, these were mitigated through triangulation with health facility or community-based surveillance data and with direct observations.

## Conclusions

This study provided an overview of the current WASH situation in Cox’s Bazar refugee camps, as well as a review of the changes that occurred since the initial emergency response in 2018. The population of this area have seen improvements in access to water over the past years but remain highly vulnerable to WASH-related diseases due to limited continuous water supply and a lack of access to soap and improved sanitation facilities. A multisectoral approach to address these needs is crucial to reduce risk and prevent further spread of WASH-related diseases in this community.

## Supplementary Information

Supplementary Material 1.

Supplementary Material 2.

## Data Availability

The dataset used and analysed during the current study are available from the corresponding author on reasonable request.

## Abbreviations

CI: Confidence interval
DR: Decision rule
LQAS: Lot Quality Assurance Sampling
LSTM: Liverpool School of Tropical Medicine
MSF: Médecins sans Frontières
SA: Supervision Area
UNHCR: United Nations High Commission for Refugees
WASH: Water, Sanitation and Hygiene
