# Supplementary material for: Assessment of water, sanitation and hygiene services within nineteen Rohingya camps in Cox’s Bazar, Bangladesh in 2022
Source: BMC Health Serv Res. 2025 Jun 9;25:814. doi: 10.1186/s12913-025-12874-8 (PMC12147237; doi:10.1186/s12913-025-12874-8)
Supplement: Supplementary file 1 — Supplementary Material 1. [file 12913_2025_12874_MOESM1_ESM.docx]

**Water, Sanitation, and Hygiene Household Questionnaire**

| **HOUSEHOLD IDENTIFICATION** |  |
| --- | --- |
| Camp | _________________________ |
| Block | _________________________ |
| Random Location (1 – 19) | _________________________ |
| Team # | _________________________ |
| Date of interview | ______ /______ /______ |
|  |  |

**_____________________________________________________________**

**DEMOGRAPHICS**

How many people slept here last night?

How many children less than 5 years old live here?

How many people more than 50 years old live here?

Who is head of household? (Circle one) MALE FEMALE

Gender of respondent? MALE FEMALE

**Section 1: Water Supply**

| **No.** | **Questions** | **Answers** | **Coding** | **Skips** |
| --- | --- | --- | --- | --- |
| W1 | **Where do you get drinking water?**  **PICK ONE**  **** | TUBE WELL  TAP STAND (PIPED DIRECT)  RAINWATER  WATER TANK  TANKER TRUCK  RIVER OR STREAM  PUDDLE OR SHALLOW HOLE  OTHER (SPECIFY)  _____________________ | 1  2  3  4  5  6  7  99 | **→ W3**  **→ W5**  **→ W5**  **→ W5**  **→ W5**  **→ W5**  **→ W5** |
| W2 | **Look at tube well and select all that apply.**   | PLAQUE CONTAINING APPROVED AGENCY NAME  CONCRETE APRON  GREATER THAN 15 STEPS FROM LATRINE | 1  2  3 | **All skip to W4** |
| W3 | **Turn Tap on and off and select all that apply**   | Can turn on and off  Water flows when tap on  Tap not leaking when off | 1  2  3 |  |
| W4 | In last week, any day no water from this place? | YES  NO | 1  2 |  |
| W5 | Is the taste of water okay FROM TAPSTAND OR TUBEWELL? | YES  NO  NEVER USED THIS SOURCE | 1  2  99 |  |
| W6 | **Where do you get water for cooking?**  **** | TUBE WELL  TAP STAND (PIPED DIRECT)  RAINWATER  WATER TANK  TANKER TRUCK  RIVER OR STREAM  PUDDLE OR SHALLOW HOLE  OTHER (SPECIFY) | 1  2  3  4  5  6  7  99 | **→ W7**  **→ W7**  **→ W7**  **→ W7**  **→ W7**  **→ W7** |
| W7 | **Where do you get water for washing dishes?**   | TUBE WELL  TAP STAND (PIPED DIRECT)  RAINWATER  WATER TANK  TANKER TRUCK  RIVER OR STREAM  PUDDLE OR SHALLOW HOLE  OTHER (SPECIFY)  _____________________ | 1  2  3  4  5  6  7  99 | **→ W8**  **→ W8**  **→ W8**  **→ W8**  **→ W8**  **→ W8** |
| W8 | **Where do you get water for washing hands?**   | TUBE WELL  TAP STAND (PIPED DIRECT)  RAINWATER  WATER TANK  TANKER TRUCK  RIVER OR STREAM  PUDDLE OR SHALLOW HOLE  OTHER (SPECIFY) | 1  2  3  4  5  6  7  99 | **→ W9**  **→ W9**  **→ W9**  **→ W9**  **→ W9**  **→ W9** |
| W9 | **Where do you get water for washing clothes?**   | TUBE WELL  TAP STAND (PIPED DIRECT)  RAINWATER  WATER TANK  TANKER TRUCK  RIVER OR STREAM  PUDDLE OR SHALLOW HOLE  OTHER (SPECIFY) | 1  2  3  4  5  6  7  99 | **→ W10**  **→ W10**  **→ W10**  **→ W10**  **→ W10**  **→ W10** |
| W10 | Do you ever take water from river, stream, puddle, hand dug well, or shallow hole? | YES  NO | 1  2 | **→ SS1** |
| W11 | **Which one do you use?**  **SELECT ALL THAT APPLY**   | RIVER (moving water)  PUDDLE (after rain)  SHALLOW HOLE (dug by community)  OTHER (SPECIFY) | 1  2  3  99 |  |
| W12 | **How do you use water from that place (answer to question W12)?**  **SELECT ALL THAT APPLY**  **** | DRINKING  COOKING  WASHING DISHES  WASHING HANDS  BATHING (MALES)  BATHING (FEMALES)  WASHING CLOTHES  OTHER (SPECIFY) | 1  2  3  4  5  6  7  99 |  |

**Section 2: Water Storage and Safety**

| **No.** | **Questions** | **Answers** | **Coding** | **Skips** |
| --- | --- | --- | --- | --- |
| SS1 | Do you keep water here? | YES  NO | 1  2 | **→ SS12** |
| SS2 | Can we see how you keep water? | YES  NO | 1  2 | **→ SS12** |
| SS3 | **Look at the water containers. Which ones do they have?**  **SELECT ALL THAT APPLY** | METAL POT  PLASTIC JUG / BUCKET  CLAY POT  JERRY CAN  DRUM / BARREL  OTHER (SPECIFY) | 1  2  3  4  5  99 |  |
| SS4 | Look and count how many water containers are in household.  | (Enter whole number) |  |  |
| SS5 | Look and select all sizes of containers. | (Enter whole number) |  |  |
| SS6a, b, c, d | HOW MANY CONTAINERS OF 8L, 10L, 12L, 20L? | Count for each size |  |  |
| SS7 | How many containers have drinking water? | (Enter whole number) |  |  |
| SS8 | CAN WE SEE YOUR DRINKING WATER CONTAINERS? |  |  |  |
| SS9 | Look and select all that describe what drinking water container looks like.  **SELECT ALL THAT APPLY** | LID OR SECURE FITTING COVER  A NARROW MOUTH (UNDER 10 CM)  CLEAN (NO VISIBLE DIRT INSIDE)  NONE OF THE ABOVE | 1  2  3  4 |  |
| SS10 | When do you clean inside of container?   | TWO OR MORE TIMES A DAY  ONE TIME A DAY  TWO TIMES A WEEK  ONE TIME A WEEK  ONE TIME A MONTH  NEVER | 1  2  3  4  5  6 |  |
| SS11 | How long is water in container?   | LESS THAN ONE DAY  ONE DAY  TWO DAYS  THREE DAYS  MORE THAN THREE DAYS | 1  2  3  4  5 |  |
| SS12 | Do you have chlorine tablets? | YES  NO | 1  2 |  |
| SS13 | Did you use chlorine tablets the last time you collected water for drinking? | YES  NO | 1  2 |  |
| SS14 | 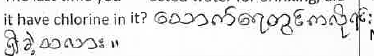The last time you collected water for drinking, did it have chlorine in it? | YES  NO | 1  2 |  |
| SS15 | Is the taste of chlorine water okay? | YES  NO  NOT SURE/ NEVER DRANK CHLORINE | 1  2  99 |  |

**Section 3: Hygiene including menstrual hygiene**

| H1 | Do you have soap?   | YES  NO | 1  2 | **→ H4** |
| --- | --- | --- | --- | --- |
| H2 | Can we see your soap?   | YES  NO | 1  2 | **→ H4** |
| H3 | Look and count how many pieces of soap in household. | (Enter whole number) |  |  |
| H4 | **Look and see if there is there water for washing in household.** | YES  NO | 1  2 |  |
| H5 | Has anyone come here to talk about hygiene? | YES  NO | 1  2 |  |
| H6 | Are there any women or girls who menstruate?  | YES  NO  DON’T KNOW | 1  2  99 | **→ SA1**  **→ SA1** |
| H7 | What do women or girls use for menstruation? | DISPOSABLE CLOTH/PAD  REUSABLE CLOTH/PAD  DON’T KNOW  OTHER (SPECIFY) | 1  2  96  99 | **→ H8**  **→ H8**  **→ H8** |
| H8 | **Where do women or girls get menstruation clothes/pads?**  | DISTRIBUTION  MARKET  HOSPITAL/CLINIC  HEALTH OR HYGIENE PROMOTER  SELF MADE  DON’T KNOW  OTHER (SPECIFY) | 1  2  3  4  5  96  99 |  |
| H9 | Where do female members of the household dispose of “single-use” menstrual hygiene products? | HOUSEHOLD LATRINE  COMMUNAL LATRINE  BURNED  COMMUNAL WASTE COLLECTION  CESS PIT  FLUSH LATRINE  SEPTIC TANK | 1  2  3  4  5  6  7 |  |

**?**

**Section 4: Latrines and Sanitation**

| SA1 | Where do male members of the household usually go to defecate?  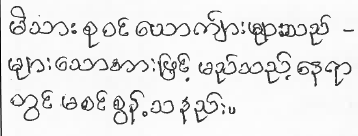**SELECT ONLY ONE RESPONSE** | HOUSEHOLD LATRINE  COMMUNAL LATRINE  BESIDE THE LATRINE  NEAR THE HOUSE  ANY OPEN SPACE  OTHER (SPECIFY) | 1  2  3  4  5  99 | **→ SA9**  **→ SA9**  **→ SA9**  **→ SA9** |
| --- | --- | --- | --- | --- |
| SA2 | WHERE IS THE NEAREST LATRINE? |  |  |  |
| SA3 | Can all men and boys use the latrine? | YES  NO | 1  2 |  |
| SA4 | Do men and boys all think this latrine is safe?  | YES  NO | 1  2 |  |
| SA5 | Look and select all that describe how men’s latrine is.  **SELECT ALL THAT APPLY**  **** | FUNCTIONAL, LOCKABLE DOOR  PLASTIC SHEETING OR NON-LOCKABLE DOOR  NO VISIBLE FAECES  COVER OVER OPENING  NOT OVERFLOWING  WITHIN 50 STEPS OF HOUSEHOLD  NONE OF THE ABOVE | 1  2  3  4  5  6  7 |  |
| SA6 | Look to see if there handwashing place at latrine.  | YES  NO | 1  2 | **→ SA9** |
| SA7 | Look to see if there is soap in handwashing area.  | YES  NO | 1  2 |  |
| SA8 | Look to see if there is water in handwashing area. | YES  NO | 1  2 |  |
| SA9 | Where do female members of the household usually go to defecate?  **SELECT ONLY ONE RESPONSE**  **** | HOUSEHOLD LATRINE  COMMUNAL LATRINE  BESIDE THE LATRINE  NEAR THE HOUSE  ANY OPEN SPACE  OTHER (SPECIFY) | 1  2  3  4  5  99 | **→ SA18**  **→ SA18**  **→ SA18**  **→ SA18** |
| SA10 | WHERE IS THE NEAREST LATRINE FOR WOMEN OR GIRLS? |  |  |  |
| SA11 | Can all women and girls use the latrine?  | YES  NO | 1  2 |  |
| SA12 | Is that latrine safe for all women and girls? | YES  NO | 1  2 |  |
| SA13 | Is that latrine also used by men or boys?  | YES  NO | 1  2 | **→ SA18** |
| SA14 | Look and select all that describe how is women’s latrine.  | FUNCTIONAL, LOCKABLE DOOR  PLASTIC SHEETING OR NON-LOCKABLE DOOR  NO VISIBLE FAECES  COVER OVER OPENING  NOT OVERFLOWING  WITHIN 50 STEPS OF HOUSEHOLD  NONE OF THE ABOVE | 1  2  3  4  5  6  7 |  |
| SA15 | Look to see if there is handwashing place at latrine. | YES  NO | 1  2 | **→ SA16** |
| SA16 | Look to see if there is soap at handwashing area. | YES  NO | 1  2 |  |
| SA17 | Look to see if there is water at handwashing area. | YES  NO | 1  2 |  |
| SA18 | Where do you throw away baby’s or children's feces?  | HOUSEHOLD LATRINE  COMMUNAL LATRINE  BESIDE THE LATRINE  NEAR THE HOUSE  ANY OPEN SPACE  NO CHILDREN  OTHER (SPECIFY) | 1  2  3  4  5  96  99 |  |

**Section 5: Solid waste management**

| SWM1 | How do you dispose of your household waste? | Communal waste collection system  Burn  Bury  Other | 1  2  3  4 | **→SWM2** |
| --- | --- | --- | --- | --- |
| SWM2 | Are you satisfied with the frequency of the communal household waste collection system? | Yes  No | 1  2 |  |
| SWM3 | Which of the following do you collect for reuse, recycling? | Cans  Paper  Plastics  Others  None of the above | 1  2  3  4  5 |  |
| SWM4 | Look to see if there is at least one 20l bucket/bin with a lid available for solid waste storage in the household | Yes  No | 1  2 |  |

**Section 6: WASH related Disease for CHILDREN UNDER 5 YEARS**

| D1 | DO YOU HAVE ANY CHILDREN LESS THAN 5 YEARS OLD? | YES  NO |  | **→End** |
| --- | --- | --- | --- | --- |
| D2 | Has child been sick in last two weeks? | YES  NO | 1  2 |  |
| D3 | What kind of sickness? | **USE D2 – D5 TO RECORD RESPONSES** |  |  |
| D4 | **MARK (DO NOT ASK):**  Mark if parent/guardian says diarrhea | YES  NO | 1  2 |  |
| D5 | **MARK (DO NOT ASK):**  Mark if parent/guardian says eye problems | YES  NO | 1  2 |  |
| D6 | **MARK (DO NOT ASK):**  Mark if parent/guardian says skin problems | YES  NO | 1  2 |  |
| D7 | **MARK (DO NOT ASK):**  Mark if parent/guardian says yellowish skin or eyes | YES  NO | 1  2 |  |
