# Supplementary material for: Assessment of water, sanitation and hygiene services within nineteen Rohingya camps in Cox’s Bazar, Bangladesh in 2022
Source: BMC Health Serv Res. 2025 Jun 9;25:814. doi: 10.1186/s12913-025-12874-8 (PMC12147237; doi:10.1186/s12913-025-12874-8)
Supplement: Supplementary file 2 — Supplementary Material 2. [file 12913_2025_12874_MOESM2_ESM.docx]

**Annex:**

**Tables and figures**

Table 1: Definitions and targets of survey indicators

| **Section** | **Indicator** | **Definition** | **Target Avg** |
| --- | --- | --- | --- |
| **Section 1: Water Supply** | Proportion of households that use an improved water source for drinking | Tube well with plaque,  concrete apron, >15 steps  to latrine; or tap stand  with functional tap; or  water tank or tanker truck | 95% |
| **Section 1: Water Supply** | Proportion of households that report the taste of the water from the nearest improved water source is acceptable | Taste of water from nearest tube well or tap stand is acceptable | 80% |
| **Section 1: Water Supply** | Proportion of households that use the same improved water source for all activities | Tube well or tap stand used for drinking, cooking, washing dishes, washing hands, male and female bathing, and washing clothes | 90% |
| **Section 1: Water Supply** | Proportion of households that DO NOT report using surface water for drinking or cooking | Does NOT use water from a river, stream, puddle, shallow hole, or other still water source for drinking | 95% |
| **Section 1: Water Supply** | Proportion of households who report that water was continuously available from their habitual water source for the last week | Access to water supply was uninterrupted in 1 week preceding the survey | 80% |
| **Section 2: Water Storage and Safety** | Proportion of households that have water containers of at least 10 litres total capacity | Water containers of at least 10 litres total capacity | 90% |
| **Section 2: Water Storage and Safety** | Proportion of households that clean the inside of water containers at least once a week | Inside of water containers cleaned at least once a week | 80% |
| **Section 2: Water Storage and Safety** | Proportion of households that keep water in household containers for less than one day | Store water in household for less than one day | 90% |
| **Section 2: Water Storage and Safety** | Proportion of households that find the taste of chlorinated water to be acceptable | Taste of chlorinated water is acceptable | 80% |
| **Section 2: Water Storage and Safety** | Proportion of households whose water was treated with chlorine, either tablet (Aquatabs) or at the point of collection when they last collected drinking water | Bucket chlorination implemented at water point at time of last drinking water collection or chlorination tablets used | 80% |
| **Section 3: Hygiene including menstrual hygiene** | Proportion of households that can show at least one piece of soap | Soap seen by interviewer | 95% |
| **Section 3: Hygiene including menstrual hygiene** | Proportion of households that currently have soap and water available for handwashing in the household | Soap and water present in household at time of interview | 90% |
| **Section 3: Hygiene including menstrual hygiene** | Proportion of households that have been visited by a hygiene promoter | Visited by hygiene promoter within the last week | 80% |
| **Section 3: Hygiene including menstrual hygiene** | Proportion of households whose female members use acceptable materials for menstrual hygiene | Reusable menstrual hygiene products are used | 95% |
| **Section 3: Hygiene including menstrual hygiene** | Proportion of households that has ever received menstrual hygiene products from a distribution | Ever received menstrual hygiene products from a distribution | 95% |
| **Section 3: Hygiene including menstrual hygiene** | Proportion of households that dispose of single use menstrual hygiene products appropriately | Disposable menstrual hygiene products disposed into household or communal latrine OR burned OR via communal waste collection, NOT into cess pit OR pour flush latrine OR septic tank | 95% |
| **Section 4: Latrines and sanitation** | Proportion of households whose male members use an improved sanitation facility with an acceptable handwashing area that has soap and water point | Male members of household use a household or communal latrine that has a handwashing area with soap and water available at time of interview, had a functional lockable door, was not overflowing or had visible feces, and was within 50 steps of the household. | 90% |
| **Section 4: Latrines and sanitation** | Proportion of households whose female members use an improved sanitation facility with an acceptable handwashing area that has soap and water point | Female members of household use a household or communal latrine that has a handwashing area with soap and water available at time of interview, had a functional lockable door, was not overflowing or had visible feces, and was within 50 steps of the household. | 90% |
| **Section 4: Latrines and sanitation** | Proportion of households that dispose of children’s and babies’ faeces in an appropriate manner | Disposal in household or communal latrine | 95% |
| **Section 5: Solid waste management** | Proportion of households that dispose of their waste via communal waste collection | Household solid waste collected via a communal waste collection system | 80% |
| **Section 5: Solid waste management** | Proportion of households that rely on other methods to get rid of their waste | Household solid waste is burned in open fire, buried, other | 60% |
| **Section 5: Solid waste management** | Proportion of household which are satisfied with the collection frequency of the waste by communal services | Waste is collected regularly, preventing littering, bad smell and vectors | 80% |
| **Section 5: Solid waste management** | Proportion of households for which specific types of waste are collected for reuse, recycling | Waste collector (formal or informal) collect cans, plastics, paper as a source of income | 50% |
| **Section 5: Solid waste management** | Proportion of households that has at least 1 bucket/garbage bin of 20l for solid waste storage | Bucket of 20l with lid present for waste disposal at time of interview | 70% |
| **Section 6: WASH related Morbidities/Disease** | Proportion of households reporting **NOT HAVING** diarrhoea among children <5 years in the last two weeks | Three or more loose or watery stools in last two weeks **NOT** reported | 80% |
| **Section 6: WASH related Morbidities/Disease** | Proportion of households reporting **NOT HAVING** eye infections among children <5 years in the last two weeks | Eye infection **NOT** reported | 80% |
| **Section 6: WASH related Morbidities/Disease** | Proportion of households reporting **NOT HAVING** skin infections among children <5 years in the last two weeks | Skin infection **NOT** reported | 80% |
| **Section 6: WASH related Morbidities/Disease** | Proportion of households reporting **NOT HAVING** jaundice among children <5 years in last two weeks | Jaundice **NOT** reported | 80% |

Table 2: Calculation of water supply indicators by supervision area, Cox’s Bazar, Bangladesh, LQAS 2022

| **Indicator** | **Number of households with positive responses by Camp out of households surveyed/Supervision area** | | | | | | | | | | | | | | | | | | | **2022** | | |
| --- | --- | --- | --- | --- | --- | --- | --- | --- | --- | --- | --- | --- | --- | --- | --- | --- | --- | --- | --- | --- | --- | --- |
| **WATER SUPPLY** | **2E** | **2W** | **6** | **7** | **9** | **10** | **8E** | **8W** | **14** | **15** | **16** | **20ext** | **11** | **12** | **13** | **17** | **18** | **19** | **20** | **Weighted average  % (DR)** | **95% CI** | **Target (DR)** |
| Proportion of households that use an improved water source for drinking | 19/19 | 19/19 | 19/19 | 19/19 | 19/19 | 19/19 | 19/19 | 18/19 | 19/19 | 19/19 | 19/19 | 19/19 | 19/19 | 18/19 | 19/19 | 19/19 | 19/19 | 19/19 | 19/19 | 99% (16) | 98%-100% | 95% (16) |
| Proportion of households that report the taste of the water from the nearest improved water source is acceptable | 18/19 | 17/19 | 17/19 | 19/19 | 16/19 | 17/19 | 18/19 | 17/19 | 16/19 | 19/19 | 18/19 | 18/19 | 17/19 | 18/19 | 18/19 | 19/19 | 19/19 | 17/19 | 19/19 | 93% (15) | 90%-96% | 80% (13) |
| Proportion of households that use the same improved water source for all activities | 19/19 | 16/19 | 19/19 | 18/19 | 18/19 | 19/19 | 18/19 | 18/19 | 15/19 | 19/19 | 18/19 | 16/19 | 10/19 | 17/19 | 17/19 | 18/19 | 19/19 | 17/19 | 8/19* | 91% (15) | 88%-93% | 90% (15) |
| Proportion of households that DO NOT report using surface water for drinking or cooking | 19/19 | 16/19 | 19/19 | 0/19* | 18/19 | 19/19 | 18/19 | 19/19 | 15/19 | 19/19 | 18/19 | 16/19 | 10/19* | 19/19 | 17/19 | 18/19 | 19/19 | 17/19 | 8/19* | 84 (13) | 82%-87% | 95% (16) |
| Proportion of households who report that water was continuously available from their habitual water source for the last week | 13/19 | 5/19 | 9/19 | 8/19 | 0/19* | 13/19 | 6/19 | 3/18* | 13/19 | 8/19 | 9/19 | 6/19 | 10/19 | 14/18 | 8/19 | 9/19 | 2/19 | 13/19 | 12/19 | 44% (5) | 39%-49% | 80% (13) |

*high priority camp

CI: Confidence interval; DR: Decision rule

Table 3: Calculation of water storage indicators by supervision area, Cox’s Bazar, Bangladesh, LQAS 2022

| **Indicator** | **Number of households with positive responses by Camp out of households surveyed/Supervision area** | | | | | | | | | | | | | | | | | | | | **2022** | | | |
| --- | --- | --- | --- | --- | --- | --- | --- | --- | --- | --- | --- | --- | --- | --- | --- | --- | --- | --- | --- | --- | --- | --- | --- | --- |
| **WATER STORAGE** | **2E** | **2W** | **6** | **7** | **9** | **10** | **8E** | **8W** | **14** | **15** | **16** | **20ext** | **11** | **12** | **13** | **17** | **18** | **19** | **20** | **Weighted average  % (DR)** | | **95% CI** | **Target (DR)** |  |
| Proportion of households that have water containers of at least 10 litres total capacity | 18/19 | 19/19 | 19/19 | 19/19 | 15/19 | 18/19 | 18/19 | 19/19 | 19/19 | 18/19 | 19/19 | 19/19 | 19/19 | 19/19 | 19/19 | 19/19 | 17/19 | 19/19 | 18/19 | 96% (19) | | 94%-99% | 90% (15) |  |
| Proportion of households that clean the inside of water containers at least once a week | 16/18 | 19/19 | 17/19 | 19/19 | 16/17 | 12/18 | 16/19 | 18/19 | 19/19 | 15/18 | 18/19 | 18/19 | 19/19 | 19/19 | 18/19 | 17/19 | 16/17 | 19/19 | 16/18 | 92% (15) | | 89%-95% | 80% (13) |  |
| Proportion of households that keep water in household containers for less than one day | 9/18‡ | 11/19 | 3/19 | 8/19 | 11/17 | 1/18* | 7/19 | 0/19* | 4/19 | 3/18 | 4/19 | 1/19* | 4/19 | 1/19* | 4/19 | 6/19 | 7/17‡ | 5/19 | 1/18* | 27% (2) | | 23%-32% | 90% (15) |  |
| Proportion of households that find the taste of chlorinated water to be acceptable | 17/19 | 11/19* | 15/19 | 13/19 | 13/19 | 13/19 | 13/19 | 17/19 | 17/19 | 11/19* | 16/19 | 14/19 | 13/19 | 11/19* | 14/19 | 13/19 | 18/19 | 17/19 | 18/19 | 74% (12) | | 70%-79% | 80% (13) |  |
| Proportion of households whose water was treated with chlorine, either tablet (Aquatabs) or at the point of collection when they last collected drinking water | 14/19 | 13/19 | 18/19 | 11/19* | 12/19 | 13/19 | 13/19 | 19/19 | 16/19 | 15/19 | 16/19 | 16/19 | 12/19 | 15/19 | 13/19 | 17/19 | 18/19 | 16/19 | 19/19 | 78% (12) | | 73%-82% | 80% (13) |  |

*high priority camp

‡ insufficient sample at SA level to ensure alpha and beta errors are below 10%

CI: Confidence interval; DR: Decision rule

Table 4: Calculation of hygiene practice indicators by supervision area, Cox’s Bazar, Bangladesh, LQAS 2022

| **Indicator** | **Number of households with positive responses by Camp out of households surveyed/Supervision area** | | | | | | | | | | | | | | | | | | | **2022** | | |
| --- | --- | --- | --- | --- | --- | --- | --- | --- | --- | --- | --- | --- | --- | --- | --- | --- | --- | --- | --- | --- | --- | --- |
| **HYGIENE** | **2E** | **2W** | **6** | **7** | **9** | **10** | **8E** | **8W** | **14** | **15** | **16** | **20ext** | **11** | **12** | **13** | **17** | **18** | **19** | **20** | **Weighted average  % (DR)** | **95% CI** | **Target (DR)** |
| Proportion of households that can show at least one piece of soap | 18/19 | 17/18 | 19/19 | 18/19 | 16/19 | 6/19* | 18/19 | 18/19 | 19/19 | 19/19 | 18/19 | 18/19 | 19/19 | 18/19 | 19/19 | 18/19 | 14/18* | 19/19 | 19/19 | 91% (15) | 89%-94% | 95% (16) |
| Proportion of households that currently have soap and water available for handwashing in the household | 17/19 | 17/18 | 19/19 | 17/19 | 16/19 | 6/19* | 17/19 | 15/19 | 18/19 | 14/19 | 15/19 | 15/19 | 19/19 | 17/19 | 17/19 | 15/19 | 12/18‡ | 19/19 | 19/19 | 83% (13) | 80%-87% | 90% (15) |
| Proportion of households that have been visited by a hygiene promoter within last week | 15/19 | 11/19* | 18/19 | 15/19 | 13/19 | 19/19 | 13/19 | 10/19* | 16/19 | 12/19 | 14/19 | 16/19 | 14/19 | 15/19 | 17/19 | 13/19 | 14/19 | 16/19 | 19/19 | 75% (12) | 71%-80% | 80% (13) |
| Proportion of households whose female members use acceptable materials for menstrual hygiene | 0/13‡ | 13/18‡ | 12/18‡ | 13/19 | 16/18 | 9/19 | 13/17 | 0/19* | 13/19 | 10/19 | 16/19 | 6/19* | 13/16 | 19/19 | 14/18 | 12/18‡ | 7/18‡ | 16/19 | 17/19 | 62% (9) | 57%-66% | 95% (16) |
| Proportion of households that has ever received menstrual hygiene products from a distribution | 10/13‡ | 18/18 | 4/18 | 17/19 | 17/18 | 15/19 | 17/17 | 19/19 | 6/19* | 19/19 | 13/19 | 18/19 | 0/16* | 0/19* | 16/18 | 18/18 | 15/18 | 19/19 | 18/19 | 74% (11) | 71%-77% | 95% (16) |
| Proportion of households that dispose of single use menstrual hygiene products appropriately | 13/13‡ | 5/5‡ | 5/6‡ | 6/6‡ | 2/2‡ | 7/10‡ | 4/4‡ | 18/19 | 2/5‡ | 9/9‡ | 2/2‡ | 11/13‡ | 3/3‡ | 0/0‡ | 4/4‡ | 6/6‡ | 11/11‡ | 3/3‡ | 1/2‡ | 87% (14) | 83%-91% | 95% (16) |

*high priority camp

‡ insufficient sample at SA level to ensure alpha and beta errors are below 10%

CI: Confidence interval; DR: Decision rule

Table 5: Calculation of sanitation indicators by supervision area, Cox’s Bazar, Bangladesh, LQAS 2022

| **Indicator** | **Number of households with positive responses by Camp out of households surveyed/Supervision area** | | | | | | | | | | | | | | | | | | | **2022** | | |
| --- | --- | --- | --- | --- | --- | --- | --- | --- | --- | --- | --- | --- | --- | --- | --- | --- | --- | --- | --- | --- | --- | --- |
| **SANITATION** | **2E** | **2W** | **6** | **7** | **9** | **10** | **8E** | **8W** | **14** | **15** | **16** | **20ext** | **11** | **12** | **13** | **17** | **18** | **19** | **20** | **Weighted average  % (DR)** | **95% CI** | **Target (DR)** |
| Proportion of households whose male members use an improved sanitation facility with an acceptable handwashing area that has soap and water point | 1/19 | 2/19 | 1/19 | 4/19 | 0/19 | 0/19 | 1/19 | 5/19 | 0/19 | 2/19 | 1/19 | 2/19 | 4/19 | 5/19 | 2/19 | 4/19 | 1/19 | 4/19 | 3/19 | 11% (NA) | 8%-15% | 90% (15) |
| Proportion of households whose female members use an improved sanitation facility with an acceptable handwashing area that has soap and water point | 1/19 | 2/19 | 1/19 | 4/19 | 0/19 | 0/19 | 1/19 | 5/19 | 0/18 | 2/19 | 1/19 | 2/19 | 3/19 | 5/19 | 2/19 | 4/19 | 1/19 | 4/19 | 3/19 | 11% (NA) | 8%-14% | 90% (15) |
| Proportion of households that dispose of children’s and babies’ faeces in an appropriate manner | 10/19* | 13/19 | 6/19* | 17/19 | 16/19 | 9/19* | 18/19 | 16/19 | 13/19 | 14/19 | 13/19 | 7/19* | 10/19* | 13/19 | 10/19* | 19/19 | 13/19 | 19/19 | 8/19* | 72% (11) | 66%-75% | 95% (16) |

*high priority camp

CI: Confidence interval; DR: Decision rule

Table 6: Calculation of solid waste management indicators by supervision area, Cox’s Bazar, Bangladesh, LQAS 2022

| **Indicator** | **Number of households with positive responses by Camp out of households surveyed/Supervision area** | | | | | | | | | | | | | | | | | | | **2022** | | |
| --- | --- | --- | --- | --- | --- | --- | --- | --- | --- | --- | --- | --- | --- | --- | --- | --- | --- | --- | --- | --- | --- | --- |
| **SOLID WASTE MANAGEMENT** | **2E** | **2W** | **6** | **7** | **9** | **10** | **8E** | **8W** | **14** | **15** | **16** | **20ext** | **11** | **12** | **13** | **17** | **18** | **19** | **20** | **Weighted average  % (DR)** | **95% CI** | **Target (DR)** |
| Proportion of households that dispose of their waste via communal waste collection | 17/19 | 19/19 | 9/19* | 15/19 | 19/19 | 19/19 | 13/19 | 18/19 | 9/19* | 19/19 | 13/19 | 19/19 | 19/19 | 18/19 | 17/19 | 13/19 | 17/19 | 19/19 | 17/19 | 86% (14) | 83%-89% | 80% (13) |
| Proportion of household which are satisfied with the collection frequency of the waste by communal services | 17/19 | 19/19 | 8/9 | 14/15 | 19/19 | 17/19 | 13/13‡ | 17/18 | 11/19* | 19/19 | 13/13‡ | 19/19 | 19/19 | 18/18 | 16/17 | 12/13‡ | 17/19 | 19/19 | 17/19 | 98% (19) | 96%-99% | 80% (13) |
| Proportion of households for which specific types of waste are collected for reuse, recycling | 17/17 | 2/19* | 8/19 | 4/19 | 4/19 | 2/19* | 2/19* | 0/19* | 10/19 | 9/19 | 7/19 | 0/19* | 2/19* | 2/19* | 7/19 | 15/19 | 4/19 | 4/19 | 19/19 | 30% (3) | 26%-34% | 50% (7) |
| Proportion of households that has at least 1 bucket/garbage bin of 20l for solid waste storage | 1/19* | 0/19* | 0/19* | 13/19 | 0/19* | 2/19 | 10/19 | 12/19 | 3/19 | 5/19 | 4/19 | 7/19 | 0/19* | 0/19* | 4/19 | 9/19 | 3/19 | 12/19 | 4/19 | 25% (2) | 21%-29% | 70% (11) |

*high priority camp

‡insufficient sample at SA level to ensure alpha and beta errors are below 10%

CI: Confidence interval; DR: Decision rule

Table 7: Water, hygiene and sanitation related disease indicators by supervision area, Cox’s Bazar, Bangladesh, LQAS 2022

| **Indicator** | **Number of households with positive responses by Camp out of households surveyed/Supervision area** | | | | | | | | | | | | | | | | | | | | **2022** | | |
| --- | --- | --- | --- | --- | --- | --- | --- | --- | --- | --- | --- | --- | --- | --- | --- | --- | --- | --- | --- | --- | --- | --- | --- |
| **DISEASE** | **2E** | **2W** | **6** | **7** | **9** | **10** | **8E** | **8W** | **14** | **15** | **16** | **20ext** | **11** | **12** | **13** | **17** | **18** | **19** | **20** | **Weighted average  % (DR)** | | **95% CI** | **Target (DR)** |
| Proportion of households reporting NOT HAVING diarrhoea among children <5 years in the last two weeks | 17/19 | 16/19 | 13/19 | 15/19 | 15/19 | 13/19 | 13/19 | 19/19 | 16/19 | 14/19 | 15/19 | 17/19 | 12/19 | 16/19 | 16/19 | 14/19 | 15/19 | 15/19 | 13/19 | 78% (12) | | 74%-83% | 80% (13) |
| Proportion of households reporting NOT HAVING eye infections among children <5 years in the last two weeks | 19/19 | 19/19 | 19/19 | 18/19 | 19/19 | 19/19 | 17/19 | 19/19 | 18/19 | 17/19 | 19/19 | 19/19 | 17/19 | 18/19 | 18/19 | 18/19 | 19/19 | 19/19 | 19/19 | 96% (16) | | 94%-99% | 80% (13) |
| Proportion of households reporting NOT HAVING skin infections among children <5 years in the last two weeks | 17/19 | 15/19 | 9/19* | 13/19 | 16/19 | 13/19 | 9/19* | 19/19 | 12/19 | 13/19 | 9/19* | 14/19 | 8/19* | 12/19 | 11/19 | 11/19 | 13/19 | 17/19 | 15/19 | 69% (11) | | 64%-73% | 80% (13) |
| Proportion of households reporting NOT HAVING acute jaundice syndrome among children <5 years in last two weeks | 19/19 | 19/19 | 19/19 | 18/19 | 19/19 | 19/19 | 18/19 | 19/19 | 19/19 | 19/19 | 19/19 | 17/19 | 19/19 | 19/19 | 16/19 | 15/19 | 19/19 | 19/19 | 19/19 | 93% (15) | | 92%-94% | 80% (13) |

*high priority camp

CI: Confidence interval; DR: Decision rule
